# Supplementary material for: Immune and spermatogenesis-related loci are involved in the development of extreme patterns of male infertility
Source: Commun Biol. 2022 Nov 10;5:1220. doi: 10.1038/s42003-022-04192-0 (PMC9649734; doi:10.1038/s42003-022-04192-0)
Supplement: Supplementary file 2 — Supplementary Information [file 42003_2022_4192_MOESM2_ESM.pdf]

Communications Biology

Supplementary Figures and Tables

Immune and spermatogenesis-related loci are involved in the development of extreme patterns of male infertility

Miriam Cerván-Martín*, Frank Tüttelmann*, Alexandra M. Lopes*, Lara Bossini-Castillo, Rocío Rivera-Egea, Nicolás Garrido, Saturnino Lujan, Gema Romeu, Samuel Santos-Ribeiro, José A. Castilla, M. Carmen Gonzalvo, Ana Clavero, Vicente Maldonado, F. Javier Vicente, Sara González-Muñoz, Andrea Guzmán-Jiménez, Miguel Burgos, Rafael Jiménez, Alberto Pacheco, Cristina González, Susana Gómez, David Amorós, Jesus Aguilar, Fernando Quintana, Carlos Calhaz-Jorge, Ana Aguiar, Joaquim Nunes, Sandra Sousa, Isabel Pereira, Maria Graça Pinto, Sónia Correia, Josvany Sánchez-Curbelo, Olga López-Rodrigo, Javier Martín, Iris Pereira-Caetano, Patricia I. Marques, Filipa Carvalho, Alberto Barros, Jörg Gromoll, Lluís Bassas, Susana Seixas, João Gonçalves, Sara Larriba, Sabine Kliesch, Rogelio J. Palomino-Morales¶, F. David Carmona¶.

* These authors contributed equally.

¶ These authors jointly supervised this work.

Supplementary Figure 1. Manhattan plot representation of the GWAS results for the discovery cohort and the meta-analysis with the replication cohort according to the different analysed subgroups. The $-\log_{10}$ of the logistic regression test P-values are plotted against its physical chromosomal position. The red line represents the genome-wide level of significance (P < 5E-08).

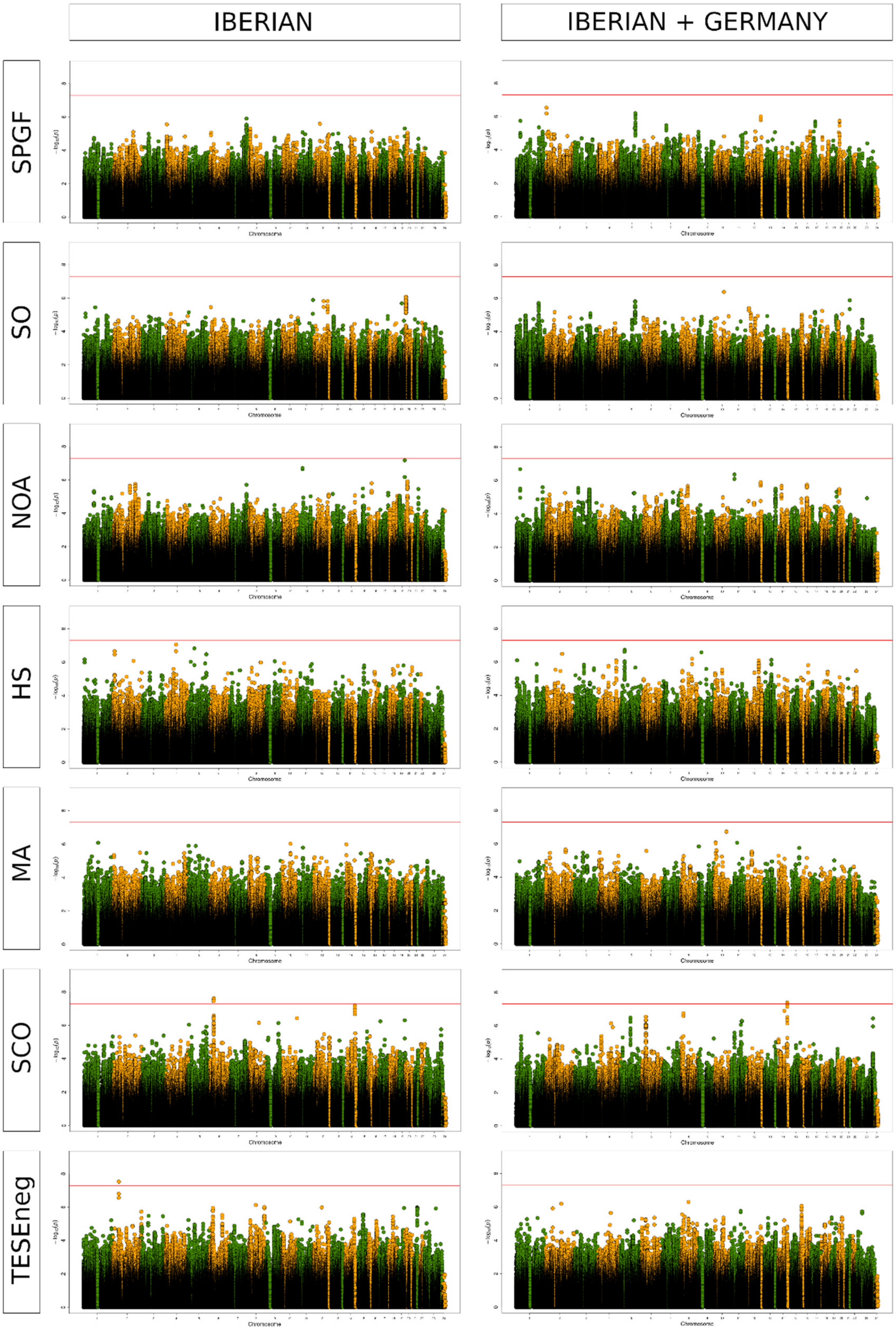

Supplementary Figure 2. Manhattan plot representation of the logistic regression test for the *FSHR* region accordingly with TESE outcome in the Iberian discovery cohort **(a)**, in the German replication cohort **(b)**, and in the combined cohort **(c)**. The -$\log_{10}$ of the P-values from the logistic regression tests and the inverse variance method are plotted against their physical chromosomal position. A red/blue colour gradient was used to represent the effect size of each analysed variant (red for risk and blue for protection). The red line represents the genome-wide level of significance (P < 5E-08).

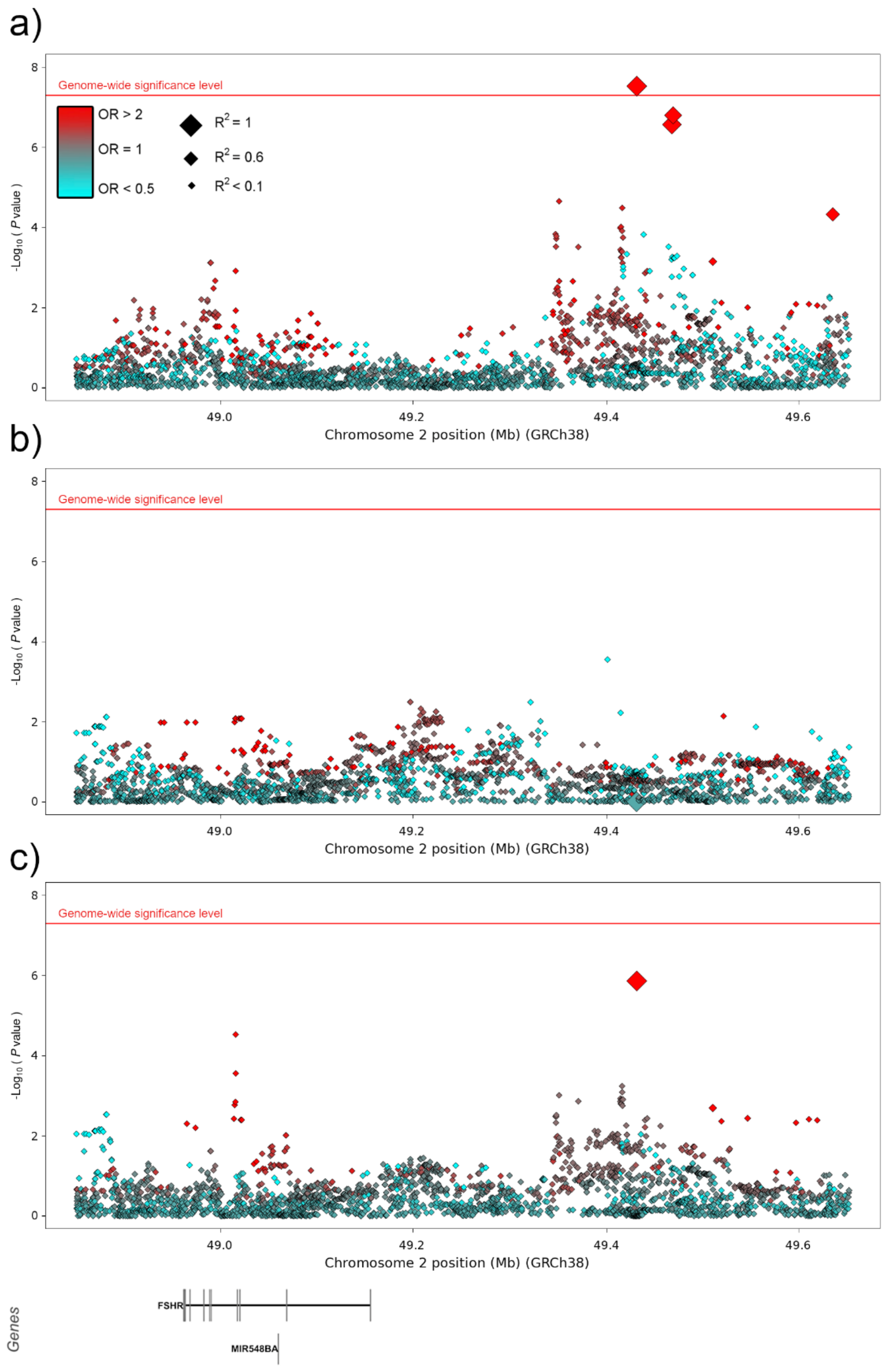

Supplementary Figure 3. Manhattan plot representation of the results from the unconditioned likelihood ratio test **(a)** and the likelihood ratio test adjusted by the position 13 of the HLA-DRβ1 molecule **(b)**. The −log10 of the combined analysis P-values are plotted against the physical chromosomal positions of the centre of codon. Each analysed MHC gene is represented with a different colour.

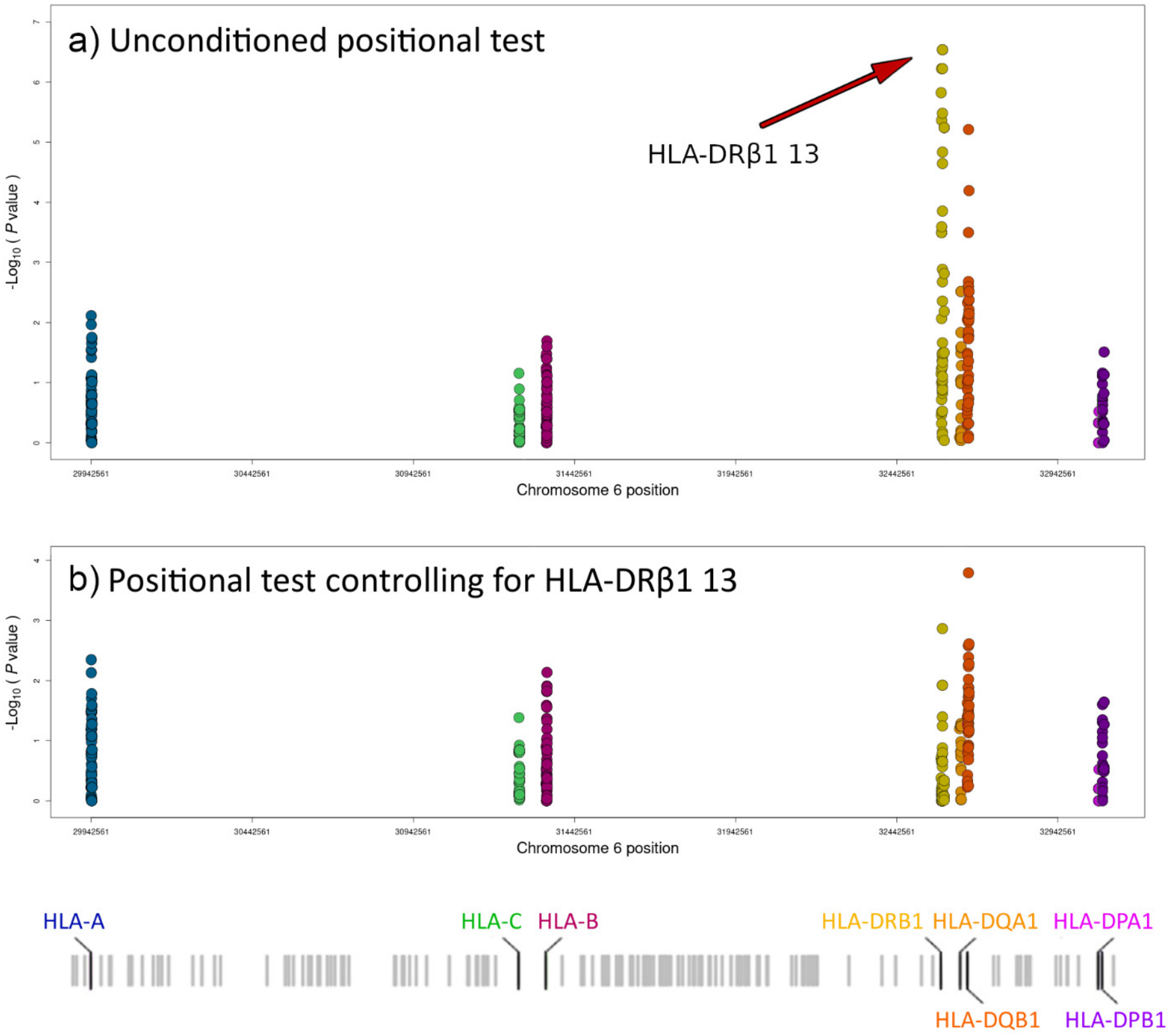

Supplementary Figure 4. Gene expression pattern of *VRK1* in human tissues. **(a)** RNA tissue specificity of the consensus dataset from the Human Protein Atlas, which consists of normalized expression (nTPM) levels for 55 tissue types, created by combining the HPA and GTEx transcriptomics datasets. Colour-coding is based on tissue groups, each consisting of tissues with functional features in common. **(b)** Testis-specific RNA expression at the single-cell level based on RNA-seq dataset from Guo *et al*. (2020), represented in a bar chart (left), according to nTPM values, and a UMAP plot (right), in which single cells are represented as coloured dots and the different colours indicate cluster identities (the intensity of the single cells correlates with the read counts). **(c)** HPA000660 antibody staining of a testis section from an unaffected 38 years-old male included in the Human Protein Atlas database (testis: T-78000; normal tissue: NOS, M-00100; patient id: 305). High expression is observed in pachytene and preleptotene spermatocytes as well as in early spermatids.

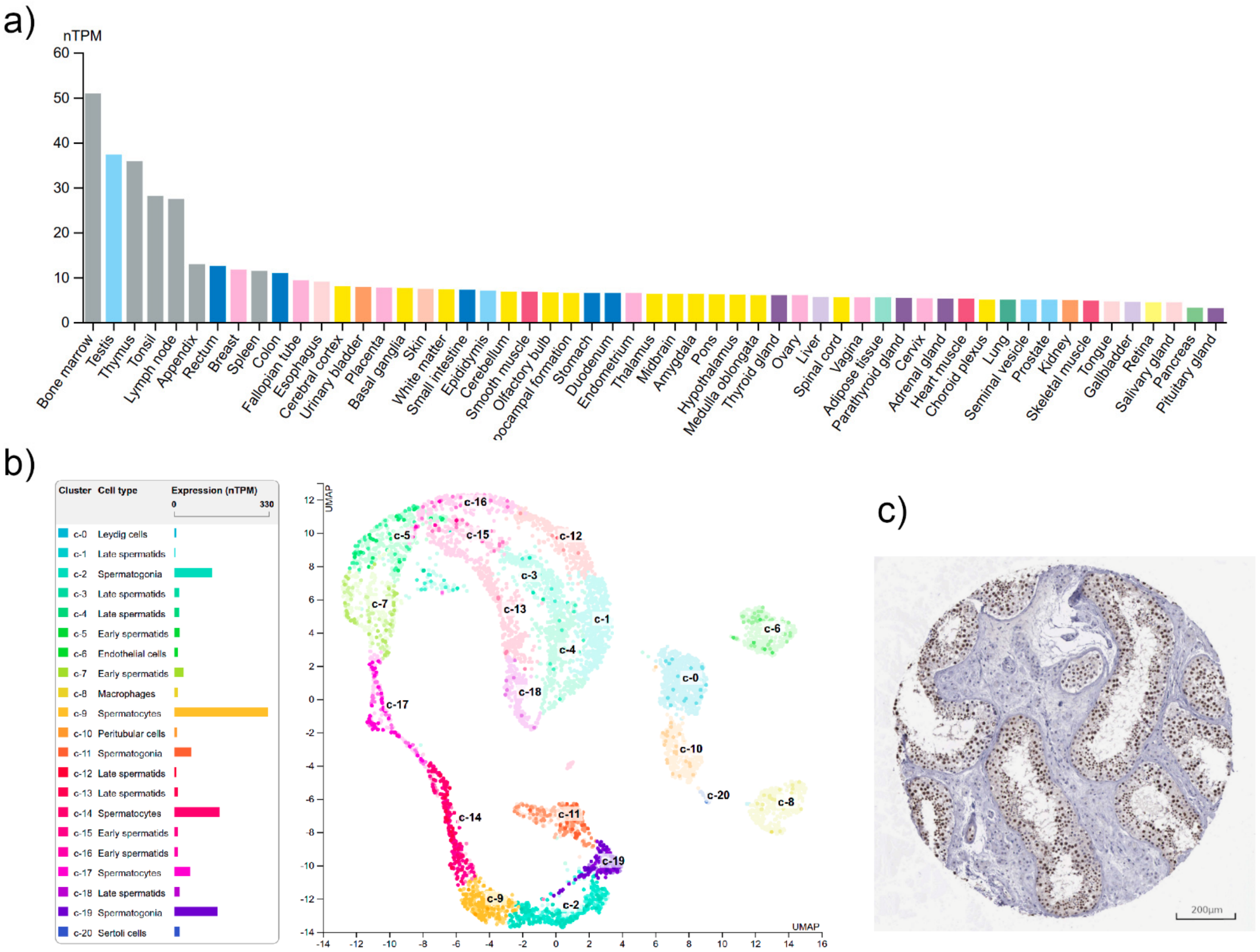

Supplementary Figure 5. GARFIELD functional enrichment analysis of the GWAS results accordingly with overall spermatogenic failure (SPGF) **(a)** and non-obstructive azoospermia (NOA) (**b**). The radial axis represents the enrichment (OR) for each of the analysed cell types that are sorted by tissue along the outside edge of the plot. Boxes forming the edge are coloured by tissue. Enrichment is calculated for the GWAS P-value threshold P < 1E-05. Dots in the inner ring of the outer circle denote significant GARFIELD enrichment (if present) after multiple-testing correction for the number of effective annotations and are coloured with respect to the tissue cell type tested (font size of tissue labels reflects the number of cell types from that tissue).

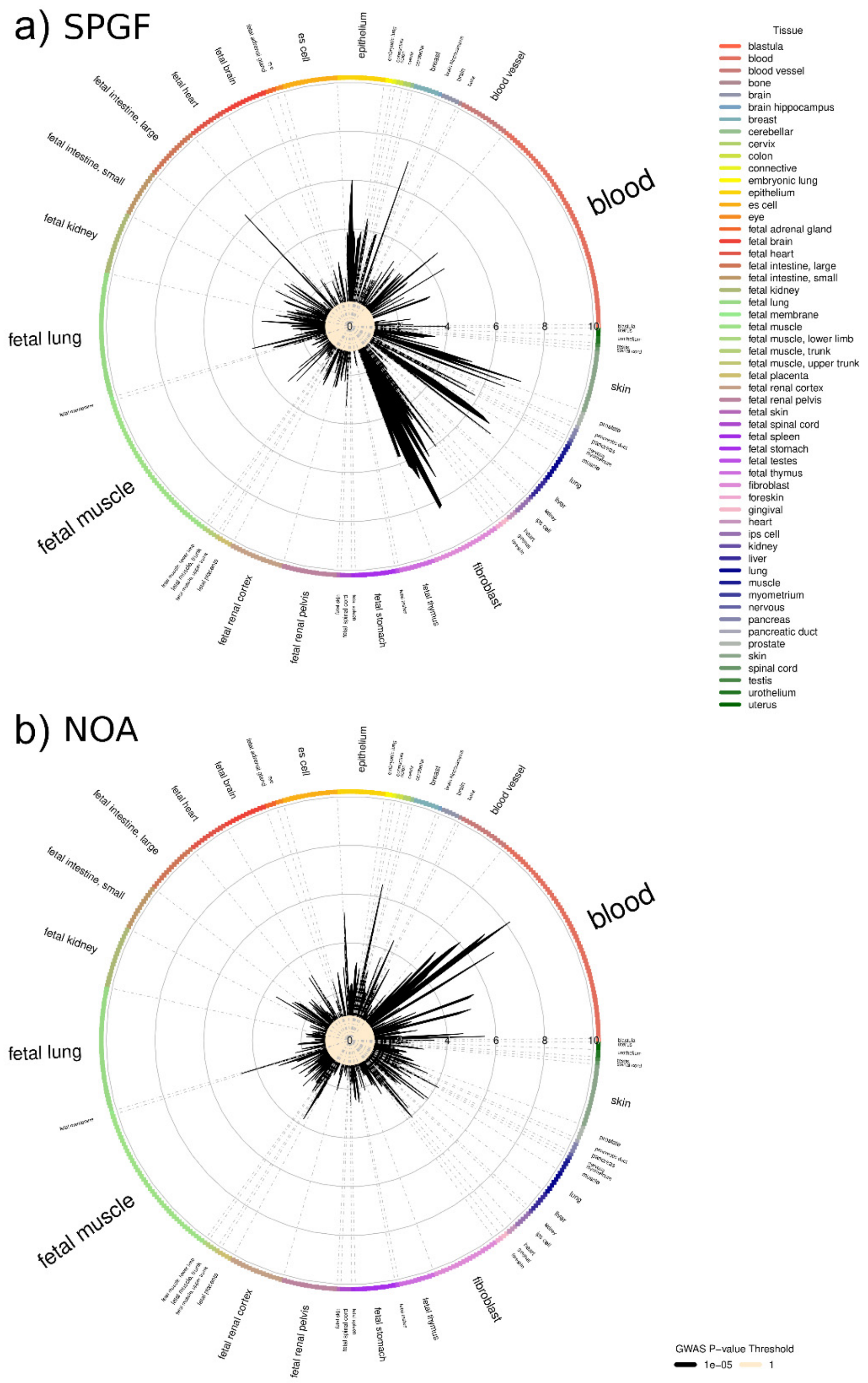

Supplementary Figure 6. GARFIELD functional enrichment analysis of the GWAS results accordingly with severe oligozoospermia (SO) **(a)** and hypospermatogenesis (HS) **(b)**. The radial axis represents the enrichment (OR) for each of the analysed cell types that are sorted by tissue along the outside edge of the plot. Boxes forming the edge are coloured by tissue. Enrichment is calculated for the GWAS P-value threshold P < 1E-05. Dots in the inner ring of the outer circle denote significant GARFIELD enrichment (if present) after multiple-testing correction for the number of effective annotations and are coloured with respect to the tissue cell type tested (font size of tissue labels reflects the number of cell types from that tissue).

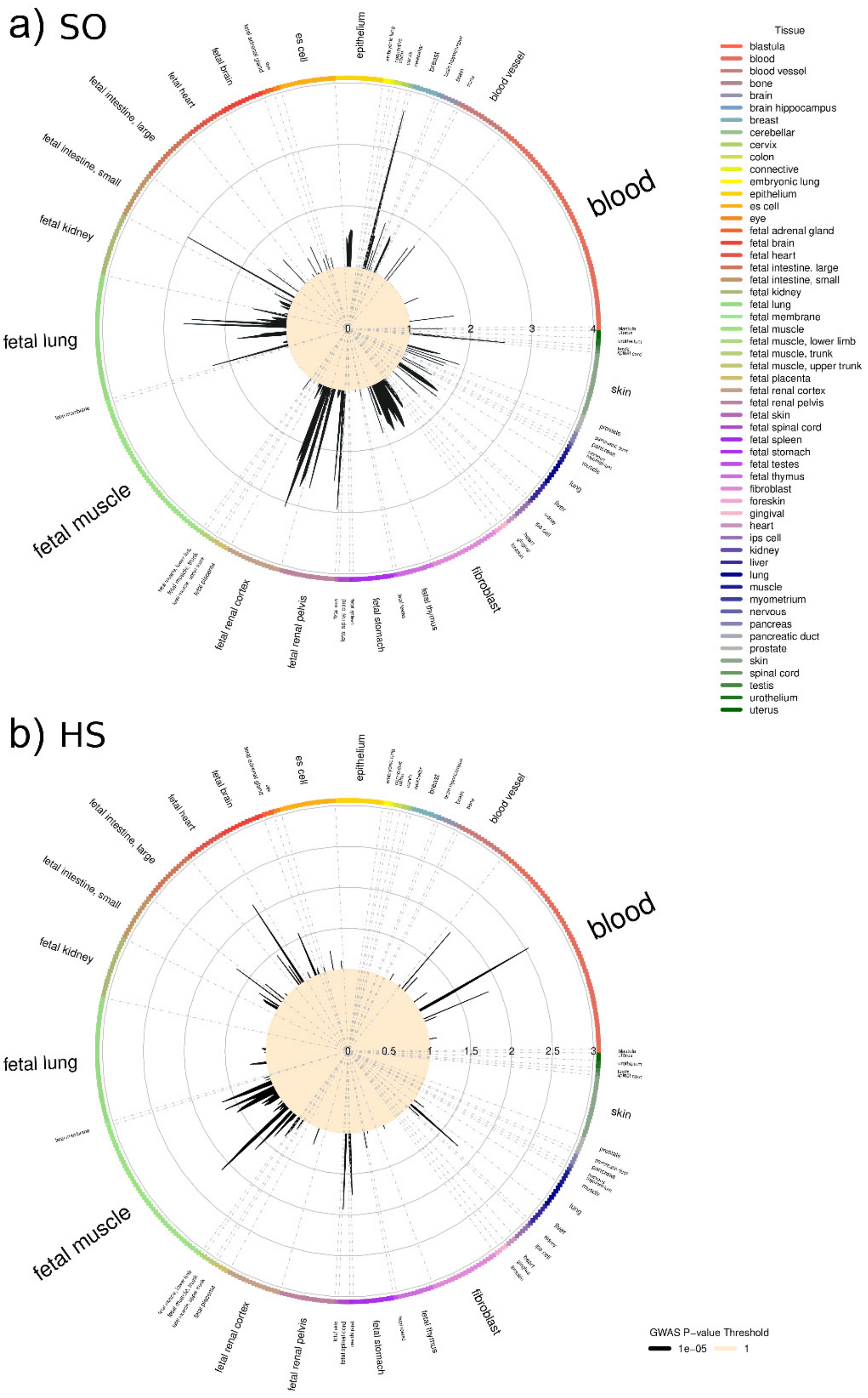

Supplementary Figure 7. GARFIELD functional enrichment analysis of the GWAS results accordingly with maturation arrest of the germ line (MA) **(a)** and unsuccessful testicular sperm extraction (TESEneg) **(b)**. The radial axis represents the enrichment (OR) for each of the analysed cell types that are sorted by tissue along the outside edge of the plot. Boxes forming the edge are coloured by tissue. Enrichment is calculated for the GWAS P-value threshold P < 1E-05. Dots in the inner ring of the outer circle denote significant GARFIELD enrichment (if present) after multiple-testing correction for the number of effective annotations and are coloured with respect to the tissue cell type tested (font size of tissue labels reflects the number of cell types from that tissue).

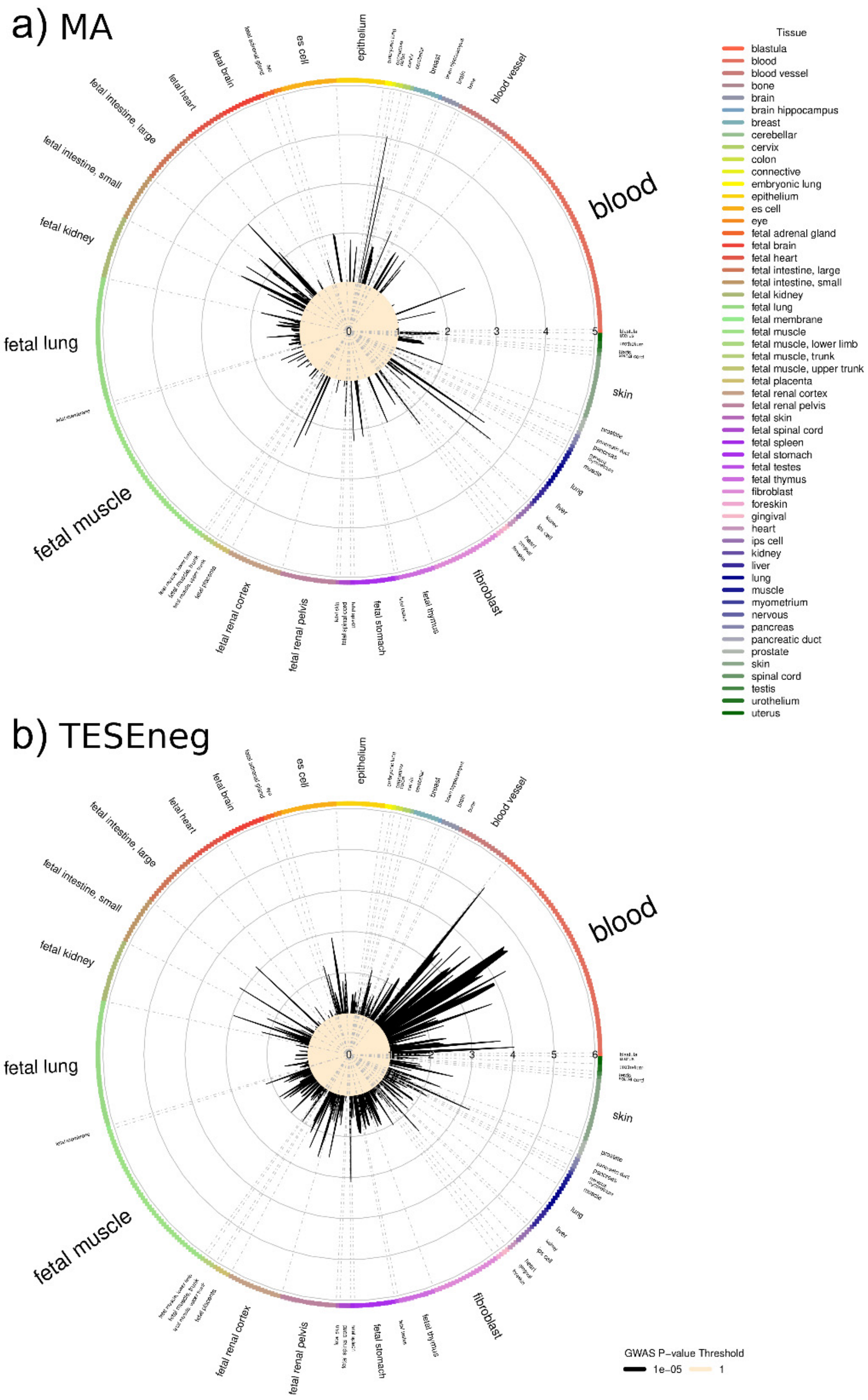

Supplementary Figure 8. Regional plots of the *NR3C1* **(a)** and *IL17A* **(b)** genomic regions in the overall meta-analysis accordingly with non-obstructive azoospermia (NOA) and Sertoli cell-only (SCO) phenotype. Lead variants are highlighted in violet.

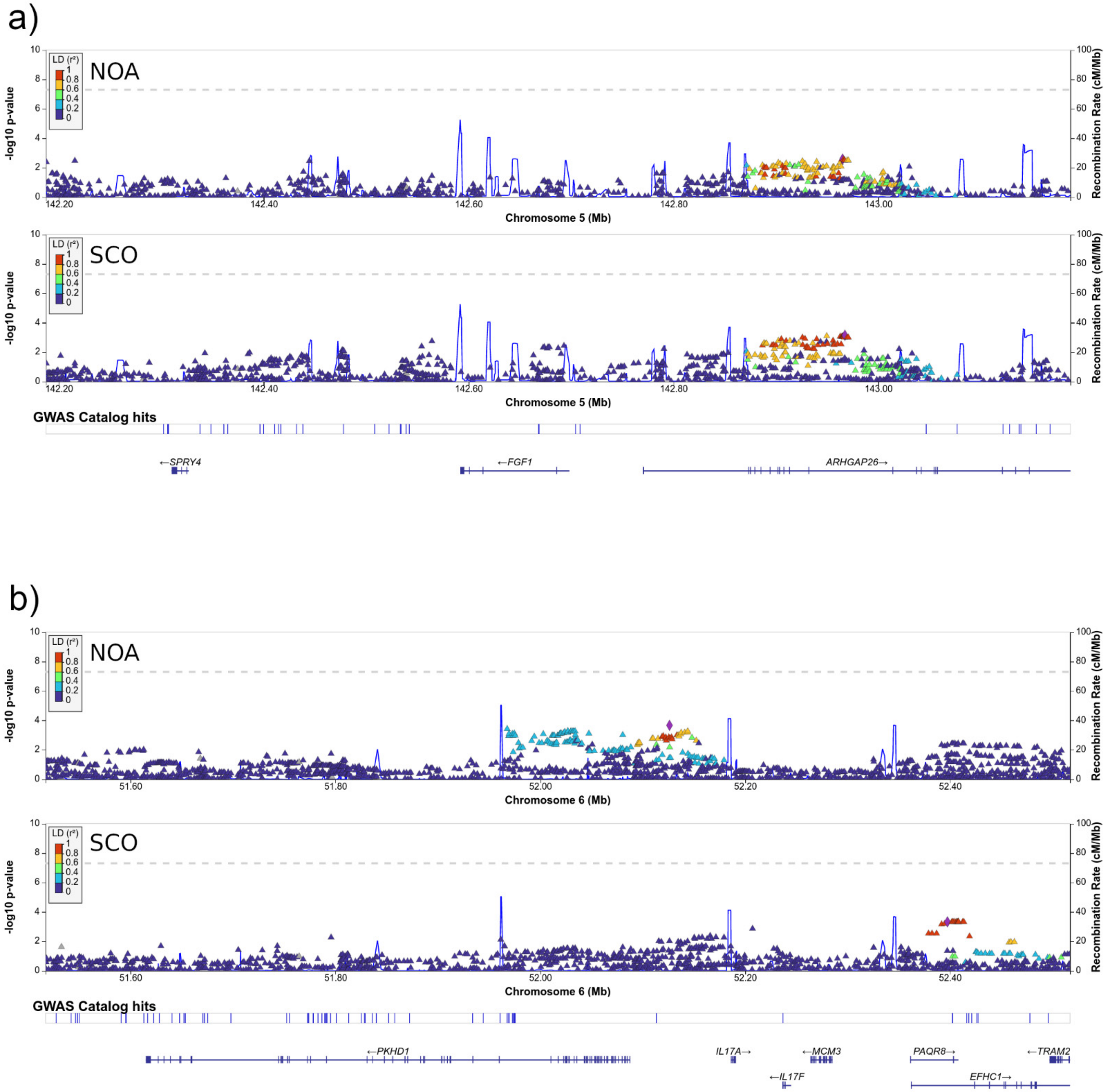

Supplementary Figure 9. Regional plots of the *PEX10* **(a)** and *PMRT6* **(b)** genomic regions in the overall meta-analysis accordingly with non-obstructive azoospermia (NOA) and Sertoli cell-only (SCO) phenotype. Lead variants are highlighted in violet.

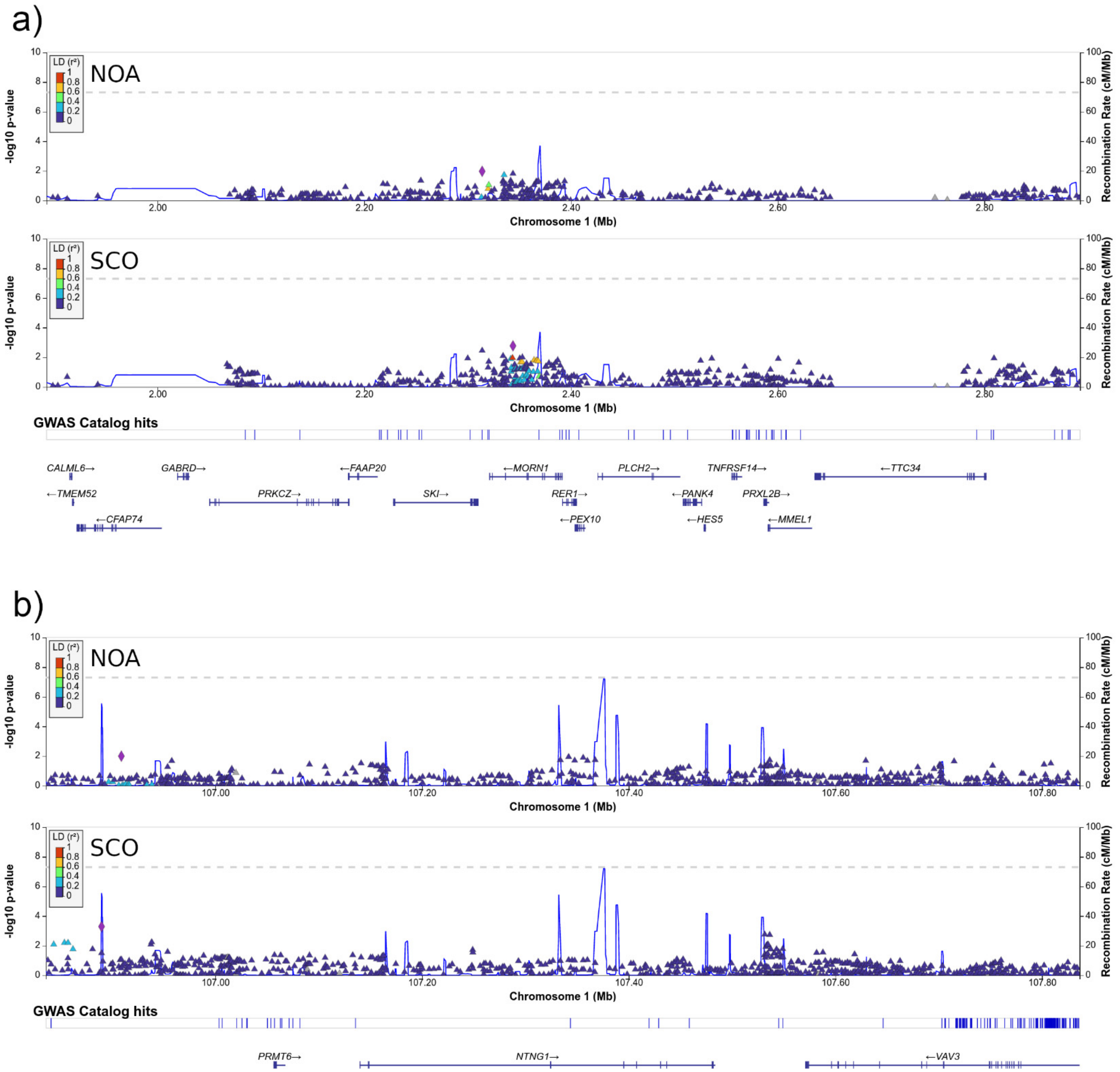

Supplementary Figure 10. Regional plots of the *ABLIM1* **(a)** and *SOX5* **(b)** genomic regions in the overall meta-analysis accordingly with non-obstructive azoospermia (NOA) and Sertoli cell-only (SCO) phenotype. Lead variants are highlighted in violet.

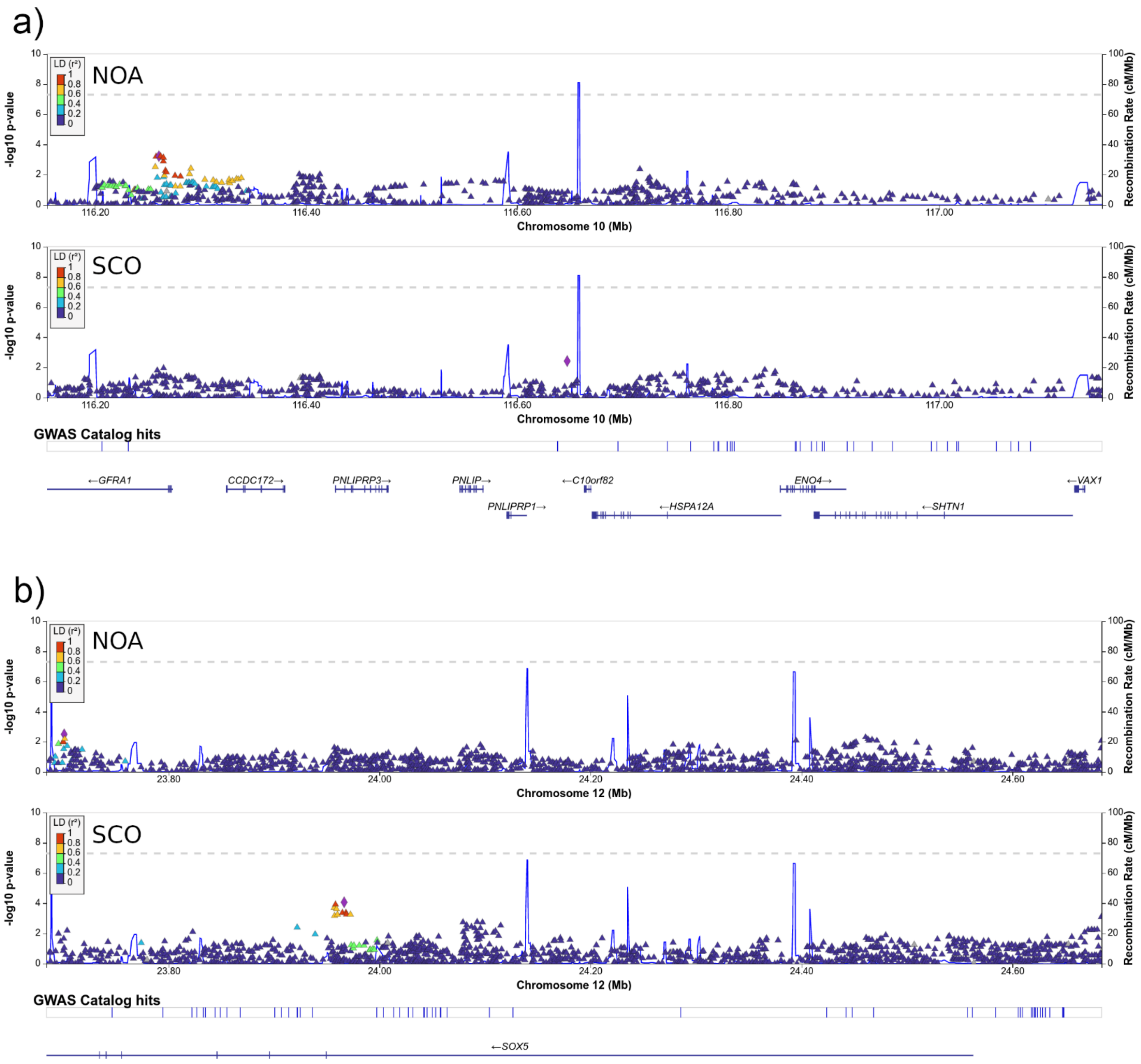

Supplementary Figure 11. Plot of the first and second principal components of the case-control cohorts analysed in this study. Cases are represented by circles and controls are represented by squares.

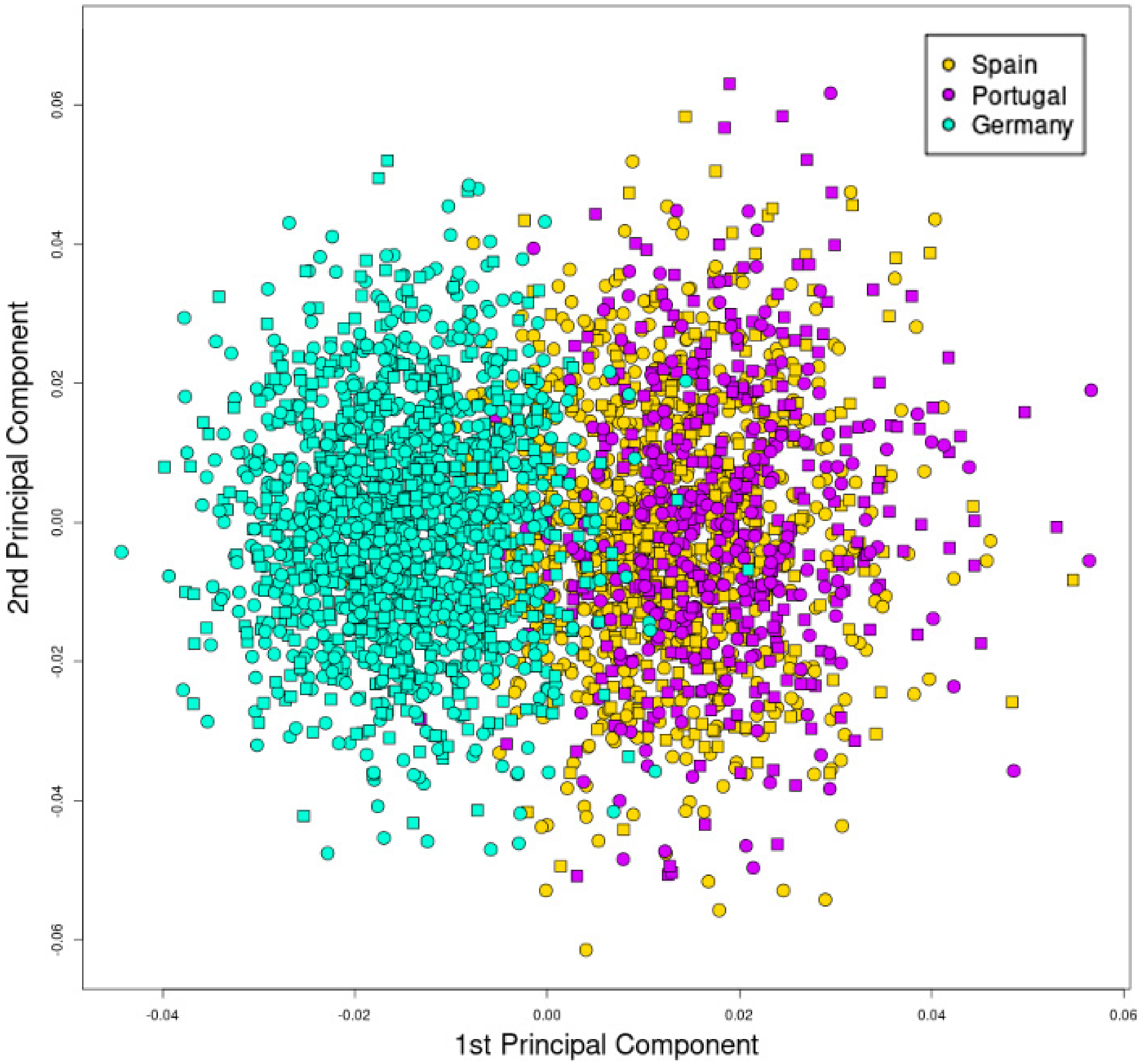

Supplementary Table 1. Main clinical features of the 1,274 infertile men included in the study. Percentages refer to all individuals with available information for the variable.

	IBERIAN (n=627)		GERMANY (n=647)	
**Clinical_feature**	**N**	**Value**	**N**	**Value**
Age at diagnosis, years	230	36 (8)*	647	34 (7)*
Severe oligozoospermia (SO)	172	27.43 %	322	49.77 %
Follicle stimulating hormone (FSH) levels, IU/L	36	8.81 (8.59)*	322	12.30 (12.20)*
Luteinizing hormone (LH) levels, IU/L	22	5.84 (3.14)*	322	4.70 (3.40)*
Non-obstructive azoospermia (NOA)	455	72.57 %	325	50.23 %
Follicle stimulating hormone (FSH) levels, IU/L	227	15.20 (16.15)*	325	20.50 (15.40)*
Luteinizing hormone (LH) levels, IU/L	184	5.60 (4.11)*	325	6.80 (4.90)*
Biopsy performed in NOA	257	56.48 %	249	76.62 %
Sertoli cell-only phenotype	104	40.47 %	110	44.18 %
Meiotic arrest	49	19.07 %	49	19.68 %
Hypospermatogenesis	43	16.73 %	77	30.92 %
Successful sperm retrieval in biopsy of NOA	90	40.72 %	94	42.53 %
Unsuccessful sperm retrieval in biopsy of NOA	131	59.28 %	127	57.47 %

*Median and interquartile range (IQR) are shown for those variables.
N: number of patients with available information.

Supplementary Table 2. Overall statistical power of the case/control study cohort according to different minor allele frequencies (MAF) and odds ratios at the significance level P<5E-08.

	**Expected odds ratios**						
**MAF**	**1.1**	**1.2**	**1.3**	**1.4**	**1.5**	**2**	
0.050	0.000	0.000	0.001	0.010	0.049	0.895	
0.100	0.000	0.001	0.014	0.106	0.355	1.000	
0.200	0.000	0.006	0.105	0.459	0.830	1.000	**Estimated power**
0.300	0.000	0.015	0.209	0.658	0.934	1.000	
0.500	0.000	0.021	0.236	0.663	0.921	1.000	

Supplementary Table 3. Tools for generating functional prediction scores and significance of their values.

Method	Predicted effect	Score range	Note
CADD	Benign to pathogenic	[1, 99]	Score above 10 is considered for potentially pathogenic variants.
fitCons	Non-functional to functional	[0, 1]	Higher scores indicating more potential for interesting genomic function.
EIGEN	Non-functional to functional	[-5, 40]	With median score of around 0, higher scores indicating more likely to be functional.
EIGEN-PC	Non-functional to functional	[-5, 100]	With median score of around 0, higher scores indicating more likely to be functional.
FATHMM	Deleterious to neutral/benign	[0, 1]	Scores above 0.5 are predicted to be deleterious. Scores close to the extremes (0 or 1) yield the highest accuracy.
GWAVA	Non-functional to functional	[0, 1]	Higher scores indicating more likely to be functional.
DeepSEA	Functional to non-functional	[0, 1]	Lower scores indicating higher likelihood of functional significance.
FunSeq2	Non-functional to functional	[0, 6]	Higher scores indicating more likely to be functional.
ReMM	Non-deleterious to deleterious	[0, 1]	Higher scores indicating higher prediction of deleteriousness.

Supplementary Table 4. RegulomeDB scoring scheme. eQTL, expression quantitative trait locus; TF, transcription factor.

**Score**	**Supporting data**
1a	eQTL + TF binding + matched TF motif + matched DNase Foprint + DNase peak.
1b	eQTL + TF binding + any motif + DNase Footprint + Dnase peak.
1c	eQTL + TF binding + matched TF motif + DNase peak.
1d	eQTL + TF binding + any motif + DNase peak.
1e	eQTL + TF binding + matched TF motif.
1f	eQTL + TF binding / DNase peak.
2a	TF binding + matched TF motif + matched DNase Footprint + DNase peak.
2b	TF binding + any motif + DNase Footprint + DNase peak.
2c	TF binding + matched TF motif + DNase peak.
3a	TF binding + any motif + DNase peak.
3b	TF binding + matched TF motif.
4	TF binding + DNase peak.
5	TF binding or DNase peak.
6	Other.
