# Supplementary material for: Immune and spermatogenesis-related loci are involved in the development of extreme patterns of male infertility
Source: Commun Biol. 2022 Nov 10;5:1220. doi: 10.1038/s42003-022-04192-0 (PMC9649734; doi:10.1038/s42003-022-04192-0)
Supplement: Supplementary file 3 — Description of Additional Supplementary Data [file 42003_2022_4192_MOESM3_ESM.docx]

**Description of Additional Supplementary Files**

**File name:** Supplementary Data 1

**Description:** Polymorphic variants within the MHC system analysed in this study after imputation with the SNP2HLA algorithm.

**File name:** Supplementary Data 2

**Description:** List of all SNPs, amino acid variants, and HLA classical alleles associated at the 5% significance level with Sertoli-cell only phenotype without conditioning in the meta-analysis by the inverse variance method.

**File name:** Supplementary Data 3

**Description:** Likelihood ratio test of amino acid positions for each independent cohort and the combined population.

**File name:** Supplementary Data 4

**Description:** Functional evidences of the expression quantitative trait loci (eQTL) and splicing quantitative trait loci (sQTL) in testis tagged by the *VRK1* lead variant rs115054029 (r 2 > 0.8 in the EUR population).

**File name:** Supplementary Data 5

**Description:** Previously reported non-MHC genetic associations with NOA at the genome-wide level of significance and their effect on NOA and SCO in our study population.

**File name:** Supplementary Data 6

**Description:** Polymorphisms with the lowest P-values in our GWAS accordingly to NOA and SCO phenotypes across the previously reported non-MHC risk loci for NOA at the genome-wide level of significance in the Asian studies.

**File name:** Supplementary Data 7

**Description:** The source data behind the plots shown in the figure 4 and the supplementary figures 5-7 of the paper.
