# Supplementary material for: Immune and spermatogenesis-related loci are involved in the development of extreme patterns of male infertility
Source: Commun Biol. 2022 Nov 10;5:1220. doi: 10.1038/s42003-022-04192-0 (PMC9649734; doi:10.1038/s42003-022-04192-0)
Supplement: Supplementary file 11 — Reporting Summary [file 42003_2022_4192_MOESM11_ESM.pdf]

nature portfolio

Corresponding author(s): F. David Carmona

Last updated by author(s): Oct 24, 2022

Reporting Summary

Nature Portfolio wishes to improve the reproducibility of the work that we publish. This form provides structure for consistency and transparency in reporting. For further information on Nature Portfolio policies, see our Editorial Policies and the Editorial Policy Checklist.

Statistics

For all statistical analyses, confirm that the following items are present in the figure legend, table legend, main text, or Methods section.

n/a	Confirmed	
☐	☒	The exact sample size (*n*) for each experimental group/condition, given as a discrete number and unit of measurement
☐	☒	A statement on whether measurements were taken from distinct samples or whether the same sample was measured repeatedly
☐	☒	The statistical test(s) used AND whether they are one- or two-sided<br>*Only common tests should be described solely by name; describe more complex techniques in the Methods section.*
☐	☒	A description of all covariates tested
☐	☒	A description of any assumptions or corrections, such as tests of normality and adjustment for multiple comparisons
☐	☒	A full description of the statistical parameters including central tendency (e.g. means) or other basic estimates (e.g. regression coefficient) AND variation (e.g. standard deviation) or associated estimates of uncertainty (e.g. confidence intervals)
☐	☒	For null hypothesis testing, the test statistic (e.g. *F*, *t*, *r*) with confidence intervals, effect sizes, degrees of freedom and *P* value noted<br>*Give P values as exact values whenever suitable.*
☒	☐	For Bayesian analysis, information on the choice of priors and Markov chain Monte Carlo settings
☐	☒	For hierarchical and complex designs, identification of the appropriate level for tests and full reporting of outcomes
☐	☒	Estimates of effect sizes (e.g. Cohen's *d*, Pearson's *r*), indicating how they were calculated

Our web collection on statistics for biologists contains articles on many of the points above.

Software and code

Policy information about availability of computer code

Data collection	No software was used for data collection
Data analysis	The software used for analyses are described and/or cited in the methods section (PLINK v.1.9, R-base, GCTA64, Eagle v.2.4., minimac4, GARFIELD)

For manuscripts utilizing custom algorithms or software that are central to the research but not yet described in published literature, software must be made available to editors and reviewers. We strongly encourage code deposition in a community repository (e.g. GitHub). See the Nature Portfolio guidelines for submitting code & software for further information.

Data

Policy information about availability of data

All manuscripts must include a data availability statement. This statement should provide the following information, where applicable:

- Accession codes, unique identifiers, or web links for publicly available datasets
- A description of any restrictions on data availability
- For clinical datasets or third party data, please ensure that the statement adheres to our policy

The summary statistics of the combined GWAS analysed here (both NOA and SO) will be available through the NHGRI-EBI GWAS Catalog (https://www.ebi.ac.uk/gwas/downloads/summary-statistics). Individual-level genotype data are not publicly available because they could compromise the privacy of participants and the informed consent. The 3D structure of the HLA-DR molecule shown in figure 1 is based on the Protein Data Bank entry 3pdo, with a direct view of the peptide-

binding groove. The source data behind the plots shown in figure 4 and supplementary figures 5-7 is included in supplementary data 7. All other data are contained either in the article file and its supplementary information or available upon reasonable request to the corresponding authors.

Human research participants

Policy information about studies involving human research participants and Sex and Gender in Research.

Reporting on sex and gender	Our study involved only male subjects, who were appropriately reported in the manuscript.
Population characteristics	We analysed a case-control population composed of infertile males (median age: 36, IQR=8) and unaffected male controls (median age: 34, IQR=7). They were not under treatment as far as we know. All the available information about the main clinical features of our study cohort is shown in Supplementary Table 1.
Recruitment	Cases were recruited in different public health centres and private fertility clinics from Spain and Portugal, and at the Centre of Reproductive Medicine and Andrology, University Hospital Münster, Germany, following comprehensive selection criteria based on the approved guidelines for the management of infertile men by the American Urological Association (AUA)/ American Society for Reproductive Medicine (ASRM), the Canadian Urological Association (CUA), and the World Health Organization (WHO, 2010). DNA from control subject was provided by the National DNA Bank Carlos III (University of Salamanca, Spain), the Institute of Molecular Pathology and Immunology of the University of Porto (IPATIMUP, Portugal), and the Centre of Reproductive Medicine and Andrology, University Hospital Münste (Germany).
Ethics oversight	The followed procedures were in accordance with the tenets of the Declaration of Helsinki and received approval by the Ethics Committee "CEIM/CEI Provincial de Granada" (Andalusia, Spain) at the session held on January 26th 2021 (approval number: 1/21). Besides, each participating centre received ethical approval and complied with the requirements of their local regulatory authorities.

Note that full information on the approval of the study protocol must also be provided in the manuscript.

Field-specific reporting

Please select the one below that is the best fit for your research. If you are not sure, read the appropriate sections before making your selection.

☒ Life sciences ☐ Behavioural & social sciences ☐ Ecological, evolutionary & environmental sciences

For a reference copy of the document with all sections, see nature.com/documents/nr-reporting-summary-flat.pdf

Life sciences study design

All studies must disclose on these points even when the disclosure is negative.

Sample size	A total of 1,274 infertile men due to spermatogenic failure of unexplained origin and 1,951 unaffected male controls were included, in order to ensure an appropriate statistical power for the case-control tests assuming an additive model and a significance threshold of p < 5x10-8.
Data exclusions	Standard GWAS quality control procedures were applied for exclusion criteria. Methods describe such criteria in detail.
Replication	After evaluating the relevance of the results of a discovery phase in which an Iberian cohort was evaluated, we analysed an independent replication cohort from Germany following the same workflow described for the discovery cohort. In relation to the association described between SCO and VRK1, it is important to note that it was not detected in the discovery phase but in the meta-analysis of both study cohorts. Consequently, additional replication studies in independent populations are definitively needed before establishing VRK1 as a firm SCO gene. This is specifically acknowledged in the manuscript.
Randomization	Not relevant to our study.
Blinding	Not relevant to our study.

Reporting for specific materials, systems and methods

We require information from authors about some types of materials, experimental systems and methods used in many studies. Here, indicate whether each material, system or method listed is relevant to your study. If you are not sure if a list item applies to your research, read the appropriate section before selecting a response.

nature portfolio | reporting summary

Materials & experimental systems

n/a	Involved in the study
☒	☐ Antibodies
☒	☐ Eukaryotic cell lines
☒	☐ Palaeontology and archaeology
☒	☐ Animals and other organisms
☒	☐ Clinical data
☒	☐ Dual use research of concern

Methods

n/a	Involved in the study
☒	☐ ChIP-seq
☒	☐ Flow cytometry
☒	☐ MRI-based neuroimaging
